# Spider dragline silk composite films doped with linear and telechelic polyalanine: Effect of polyalanine on the structure and mechanical properties

**DOI:** 10.1038/s41598-018-21970-1

**Published:** 2018-02-26

**Authors:** Kousuke Tsuchiya, Takaoki Ishii, Hiroyasu Masunaga, Keiji Numata

**Affiliations:** 1 Enzyme Research Team, RIKEN Center for Sustainable Resource Science, 2-1 Hirosawa, Wako-shi, Saitama 351-0198 Japan; 2 Japan Synchrotron Radiation Research Institute, 1-1-1, Kouto, Sayo-cho, Sayo-gun, Hyogo 679-5198 Japan

## Abstract

Spider dragline silks have attracted intensive attention as eco-friendly tough materials because of their excellent mechanical property and biomass-based origin. Composite films based on a recombinant spider dragline silk protein (ADF3) from *Araneus diadematus* were prepared by doping with linear or telechelic poly(l-alanine) (L- or T-polyA, respectively) as a reinforcing agent. Higher tensile strength and toughness of the composite films were achieved with the addition of polyA compared with the tensile strength and toughness of the silk-only film. The difference in the reinforcing behavior between L- and T-polyA was associated with their primary structures, which were revealed by wide angle X-ray diffraction analysis. L-polyA showed a tendency to aggregate in the composite films and induce crystallization of the inherent silk β-sheet to afford rigid but brittle films. By contrast, T-polyA dispersion in the composite films led to the formation of β-sheet crystal of both T-polyA and the inherent silk, which imparted high strength and toughness to the silk films.

## Introduction

Natural silk fibers are composite materials composed mainly of multiple proteins along with other minor components such as lipids, glycoproteins, and inorganic salts^1,2^. These components construct the higher-order structures and impart desired functionality to the silk fiber, although the role of the minor components has not yet been clarified. Introducing additives with a sophisticated molecular design into the artificial silk materials can regulate their structure and enhance their physical properties. Recently, we demonstrated the fabrication of composite films of silk fibroin from *Bombyx mori* silkworm cocoons by the addition of telechelic-type poly(l-alanine) (T-polyA) as a reinforcing agent. The resulting composite films showed higher tensile strength than silk-only films after prestretching treatment^3^. Wide-angle X-ray diffraction (WAXD) analysis of the silk composite films revealed that the origin of the tensile reinforcement was associated with the alignment of the β-sheet crystals of both doped T-polyA and GAGAGX (mainly X = S or Y) domains. The silk materials reinforced by polypeptide additives composes of all bio-based components and are promising as sustainable alternative materials in various applications requiring high mechanical property.

Spider silk is an attractive biomass-based fibrous material that exhibits excellent biodegradable and physical properties. The Various silk proteins are created by spiders for diverse purposes. Among these silk proteins, dragline silk exhibits high strength comparable to that of steel fiber and good extensibility^1,4–6^. This feature combined with high ductility imparts spider dragline silk with the highest toughness among natural and synthetic fibers. The dragline silk proteins compose of a long repetitive middle domain and highly conserved N- and C-terminal domains, and these assemble into higher-order structures to give the silk fiber high tensile strength and toughness. The middle repetitive domain forms hard β-sheet crystallites, whereas the hydrophilic N- and C-terminal domains play an important role for the construction of oriented higher-order structures during spinning process. Until now, various biomass-based polymers such as bio-polyesters and bio-polyamides have been developed for alternative materials of those manufactured from fossil fuel^7–10^. However, these bio-based polymers show relatively low mechanical properties, which limits their usage to materials requiring high mechanical property. Spider silk-based materials can be a green candidate for replacing high engineering plastics with high strength and toughness. Unlike silkworms, spider’s cannibalistic nature hampers practical cultivation of natural spiders, and the spider silk production amount per one spider is quite low. Therefore, recombinant silk materials have been extensively studied not only to elucidate the mechanism responsible for an extraordinary mechanical property in nature but also to exploit the spider dragline silk for various applications, including bioengineered and structural materials^11–20^. Achieving physical properties comparable to those of natural spider silks is still challenging for recombinant spider silk materials. Scheibel *et al*. demonstrated that recombinant spider silk fiber shows excellent toughness as high as that of the natural silk fiber, although the profile of the stress-strain curve indicated greater ductility^21^. These recombinant spider silk materials consist of a partial repetitive sequence or a combination of the repetitive domain and terminal domain(s) extracted from the whole sequence of spider silk proteins. Films have also been prepared by molding or spin-coating of recombinant spider silk solutions in an aqueous buffer or 1,1,1,3,3,3-hexafluoro-2-propanol (HFIP)^14,22,23^.

In this work, the recombinant protein of major ampullate spidroin 2 (MaSp2) from *Araneus diadematus* (ADF3) was used to fabricate composite films doped with polyalanine derivatives. We selected one of the dragline silk proteins, ADF3, to achieve high mechanical strength and toughness for the composite films. Two types of polyA dopants—a conventional linear polyalanine (L-polyA) and T-polyA—were synthesized by chemoenzymatic polymerization using papain according to a previously reported procedure^24^, and they were used to reinforce the tensile strength and toughness of the spider silk films. The silkworm silk used in the previous work possessed GAGAGX sequences to form β-sheet crystals, whereas the polyalanine sequences crystallized into β-sheets in the spider silk materials. Therefore, polyA additives are expected to show higher miscibility with spider silk backbone than silkworm silk. We hypothesize polyA additives can homogeneously disperse in the silk composite films and cocrystallize into β-sheets with native crystalline sequence in the spider silk. The relationship between the mechanical properties and the secondary structures of the composite films was investigated by tensile deformation tests and WAXD analysis.

## Results and Discussion

**Figure 1:**
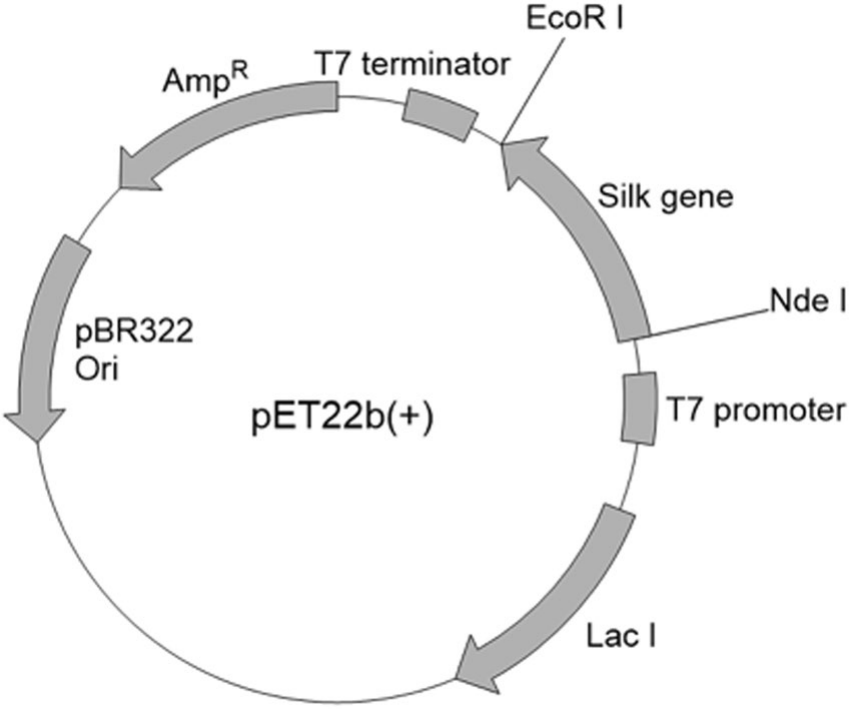
Detailed schematic illustration of plasmid DNA construct on a pET22b(+) expression vector.

**Figure 2:**
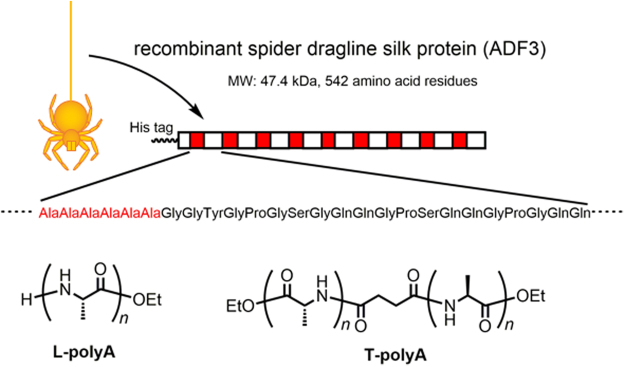
A part of the characteristic repetitive sequence of recombinant spider dragline silk based on ADF3 with a His tag at the N-terminus; the chemical structures of L-polyA and T-polyA additives are also shown.

**Figure 3:**
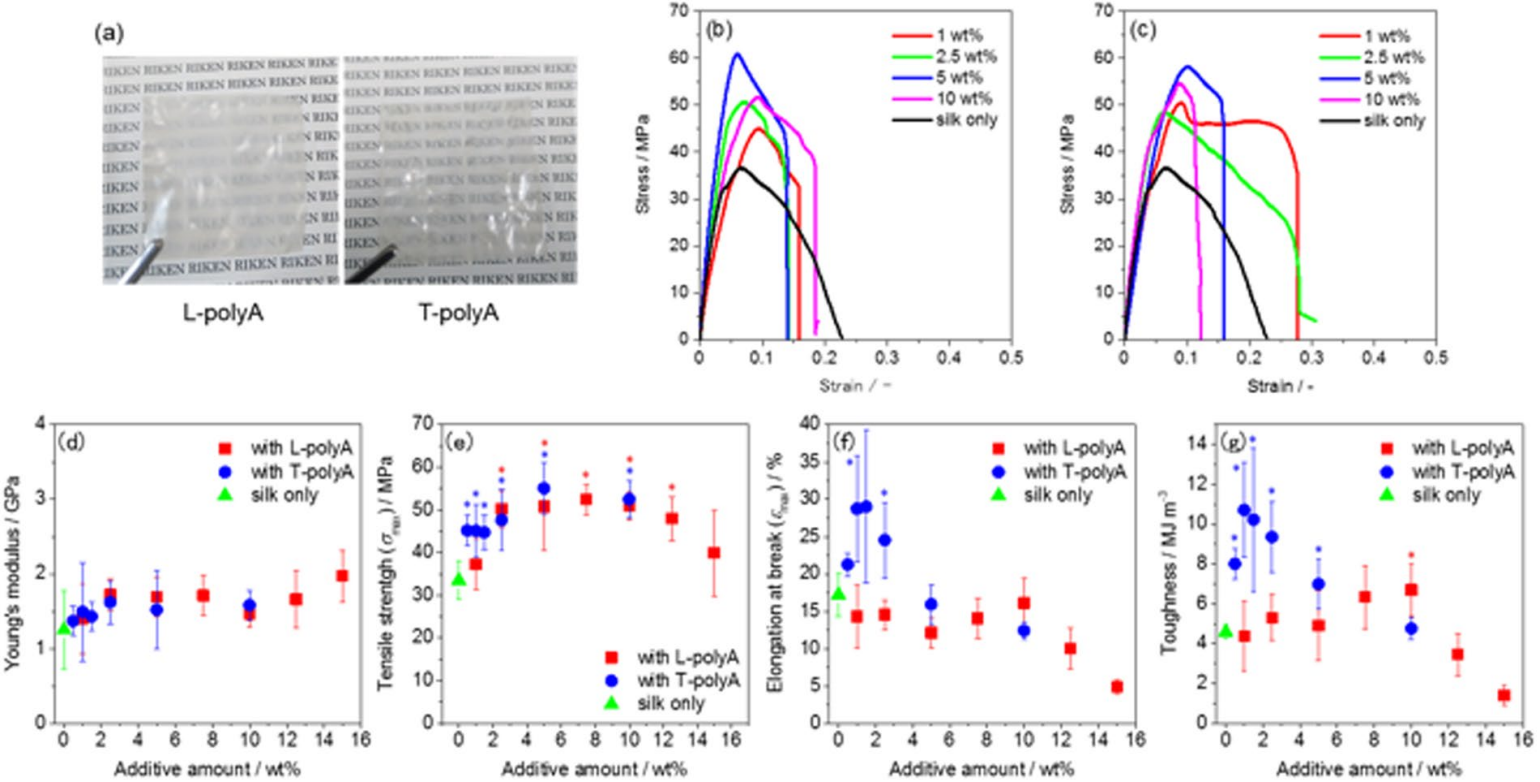
A Spider silk composite films doped with L- and T-polyA (**a**, left: 5 wt% L-polyA; right: 5 wt% T-polyA), representative stress-strain curves of the composite films doped with (**b**) L-polyA and (**c**) T-polyA with different additive amounts, and the mechanical properties of the composite films plotted against the additive amount: (**d**) Young’s modulus, (**e**) maximum tensile strength, (**f**) elongation at break, and (**g**) toughness. Each experiment was replicated 5 times and averaged to determine the standard deviation represented by the error bars. The asterisk indicates statistical significance in differences vs the silk only film (*p* < 0.05).

The recombinant spider silk protein was synthesized in *Escherichia coli* Rosetta (DE3) using pET22b(+) vector (Fig. 1). All the amino acid sequence of the recombinant silk protein is also shown in Fig. S1 in Supplementary Information. The protein was composed of a part of the repetitive domain of ADF3 and a His tag without the N- and C-terminal domains (Fig. 2) and was purified by nickel affinity chromatography. PolyA sequences periodically exist and assemble into β-sheet crystals in the repetitive domain; these β-sheet crystals impart mechanical strength to the spider silk fiber. L- and T-polyA were chemoenzymatically synthesized for use as dopants^25^. The average degrees of polymerization (DP) of L- and T-polyA were 5.8 and 5.9, respectively; these values are comparable to the polyA sequence length in ADF3 (5 to 7). The composite silk films were prepared by casting a solution of the recombinant silk protein and the polyA dopant (with a composition ranging from 0.5 to 15 wt%) in HFIP. The composite film with T-polyA was transparent, whereas that with L-polyA was slightly turbid. This difference indicates that T-polyA exhibits greater miscibility with the spider silk proteins than L-polyA (Fig. 3a). The films were immersed in methanol and subjected to prestretching with a stretching ratio ranging from 25 to 100% to induce β-sheet crystallization. Prior to the tensile tests under controlled humidity conditions, the stretched films were completely dried to exclude the effect of residual water in the films.

**Figure 4:**
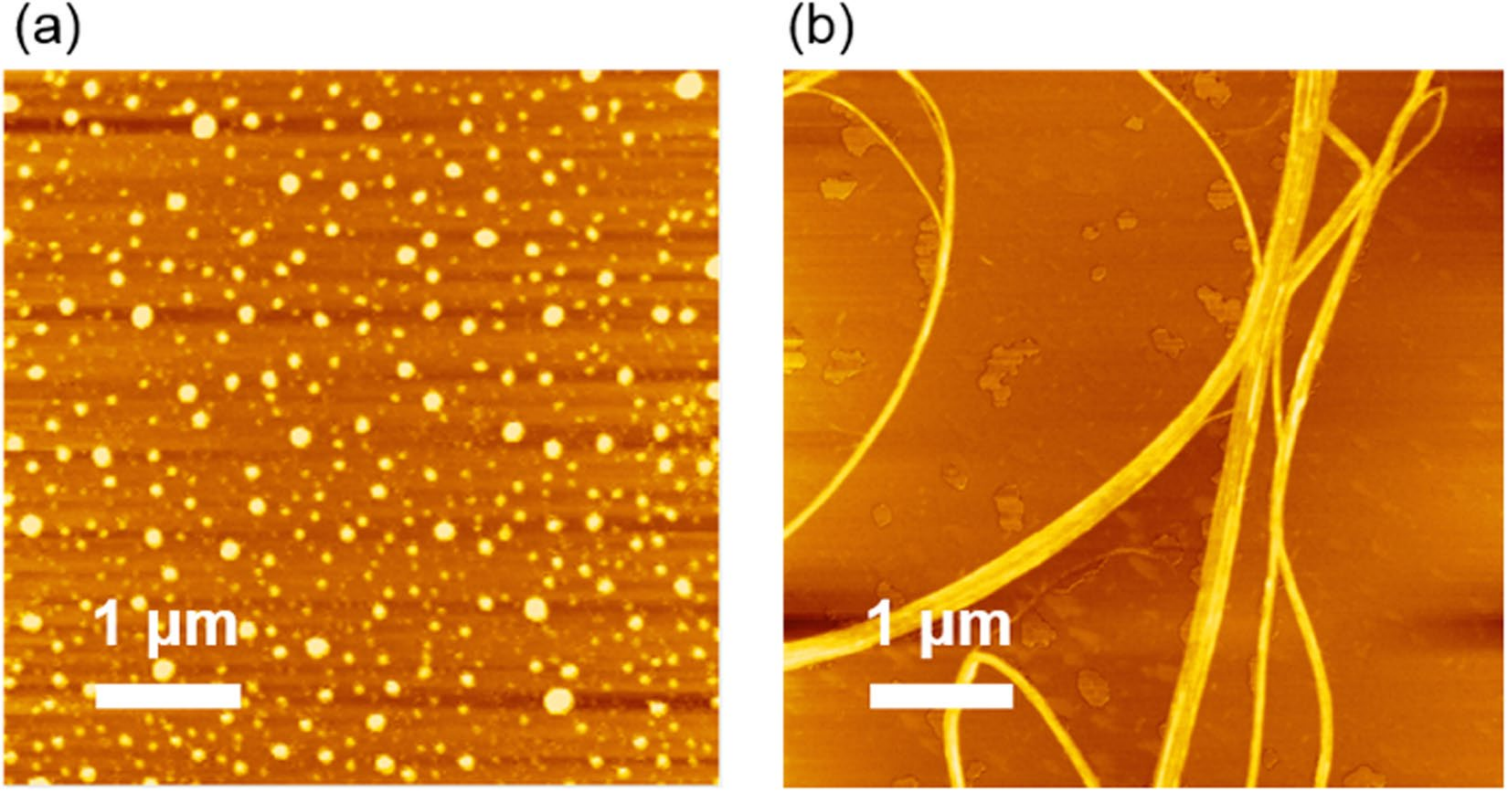
AFM topographic images of the crystals prepared from (**a**) L-polyA and (**b**) T-polyA. White bar indicates 1 μm.

The morphological difference in the self-assembly behavior between L- and T-polyA was observed by atomic force microscopy (AFM). The AFM topographic images are shown in Fig. 4. L-polyA formed granule-like crystals, whereas T-polyA adopted long fibrillar crystals with a higher aspect ratio even at a similar molecular weight. Average width and height of the single fibers were 137 ± 42 and 2.1 ± 0.73 nm, respectively, and the several fibers assembled into a thick bundle. A similar tendency was observed in a previous report^24^. The nanofiber formation of T-polyA were achieved by the unique primary structure with two different N to C direction of polyalanine backbone. The telechelic structures can assemble into two-dimensional antiparallel β-sheet structure compared to L-polyA, which probably resulted in long fibrillar formation. This difference in the crystallizing behavior is assumed to affect the secondary structure in the spider silk film and the mechanical properties.

The mechanical properties of the spider silk composite films were investigated on the basis of tensile deformation. The stress-strain curves obtained using the composite films with various prestretching ratios revealed that the tensile strength and toughness gradually increased with increasing prestretching ratio (Figs S3 and S4). In particular, when the stretching ratios of the composite films with T-polyA (5 wt%) exceeded 75%, their tensile strength became substantially greater than that of the silk-only films. According to this result and to the results in a previous report^3^, we applied a 100% prestretching ratio to further investigate the tensile behavior. In general, the mechanical properties of silk materials are known to be affected by water that interacts with the protein backbone via hydrogen bonds^26,27^. In the present work, the silk films were highly rigid in the dried state and became softer with a high extensibility and toughness at high relative humidity (RH), as evident in a series of stress-strain curves at different RHs ranging from 23 to 84% (Figs S5 and S6). At higher RH, the silk protein was more hydrated in the amorphous region and water molecules plasticized the silk film, resulting in high ductility, as reported in a previous study^26^. However, such excess hydration deteriorates the strength of silk materials and makes them difficult to handle for practical use. Thus, the relative humidity was maintained at 58% for the subsequent tensile deformation tests.

The effect of polyA as an additive on the mechanical properties of the spider silk film was examined under controlled conditions (prestretching ratio: 100%; RH: 58%). Typical stress-strain curves are shown in Fig. 3b and c, and the characteristic values are summarized in Fig. 3d–g. All of the stress-strain curves are collected in Figs S7 and S8. The Young’s moduli of the composite films were comparable to that of the original spider silk film (Fig. 3d). The addition of polyA only slightly affected the inherent β-sheet crystal structures; specifically, the presence of polyA increased the hardness of the spider silk materials. The maximum tensile strength (*σ*_max_) was enhanced by the addition of both L- and T-polyA in various compositions ranging from 0.5 to 12.5 wt% (Fig. 3e). By contrast, a different tendency was observed for the effects on the elongation at break (*ε*_max_) and toughness (Fig. 3f and g). The *ε*_max_ of the T-polyA-doped composite film reached a maximum at a T-polyA content of 1 wt% with an *ε*_max_ value approximately twice that of silk-only film, and decreased when the T-polyA content was greater than 5 wt%. By contrast, the *ε*_max_ of the L-polyA-doped composite films was comparable to that of the silk-only film and decreased when the L-polyA content exceeded 12.5 wt%. As a result, the toughness of the composite film containing 1 wt% of T-polyA was 2.5-fold greater than that of the silk-only film. Compared to the silk-only film, the composite film with L-polyA showed similar or slightly higher toughness. When the composition of T-polyA was higher than 10 wt%, the silk composite films became too brittle to obtain a self-standing film. Therefore, we could not perform tensile deformation test on the composite films with 12.5 and 15 wt% of T-polyA.

**Figure 5:**
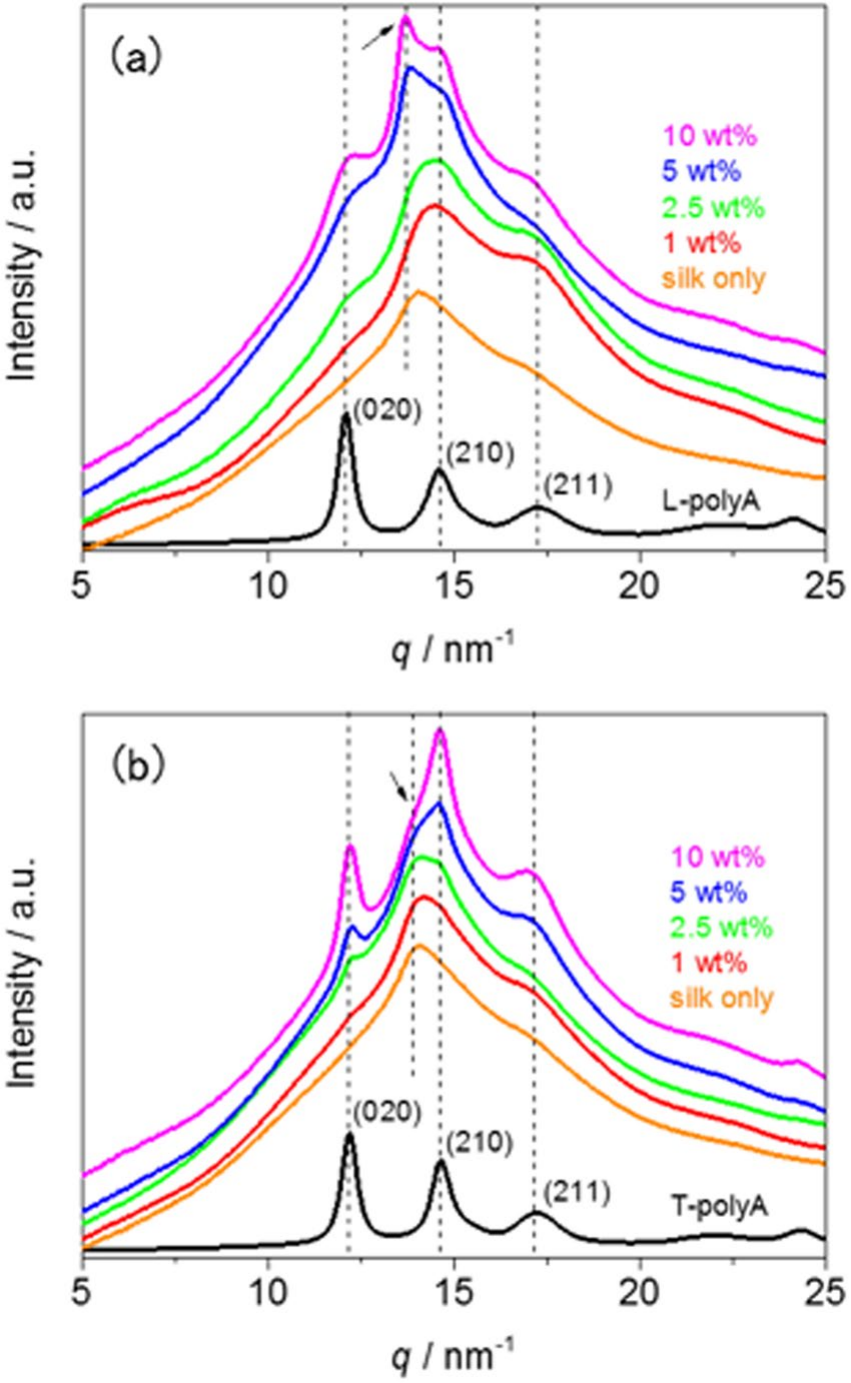
WAXD 1D profiles of silk composite films doped with (**a**) L-polyA and (**b**) T-polyA with different additive amounts (orange: silk only; red: 1 wt%; green: 2.5 wt%; blue: 5 wt%; magenta: 10 wt%). Black line shows the WAXD profile of powdered L- and T-polyA samples, with Miller indices based on a double *a*-axis for poly(l-alanine)^27,28^. Arrow shows the (210) plane of the β-sheet crystal in MaSp2 of *A. diadematus*^21^.

**Figure 6:**
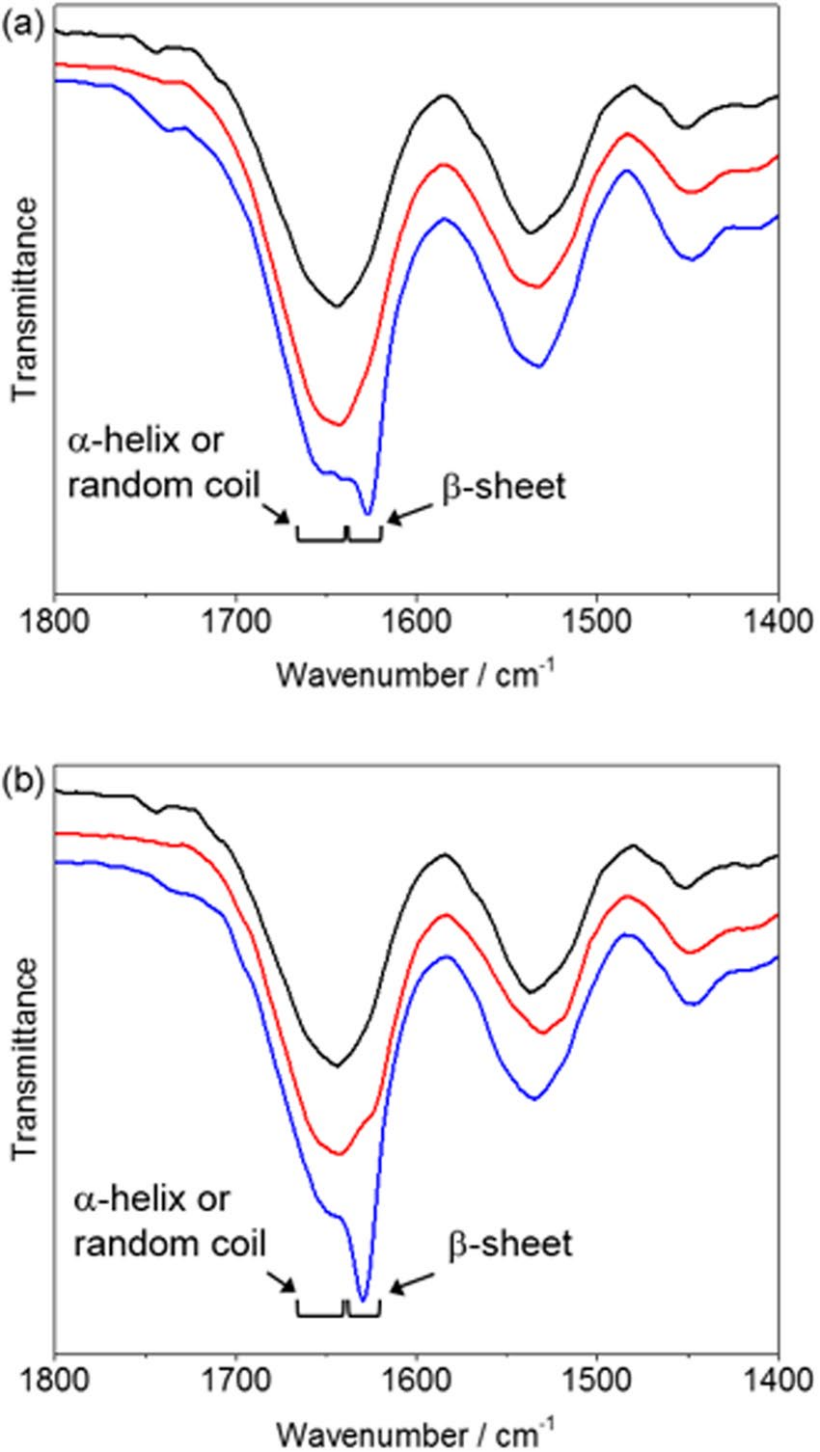
IR spectra of the silk composite films doped with (**a**) L-polyA and (**b**) T-polyA with different additive amount (black: silk only, red: 1 wt%, blue: 10 wt%).

To elucidate the mechanism of the reinforcing effect using polyA as an additive, the structures of the composite silk films were characterized by synchrotron WAXD and infrared (IR) spectroscopy analysis. The WAXD 1D profiles of the 100%-prestretched composite films with various polyA compositions are shown in Fig. 5. The profile of the silk-only film shows broad peaks derived from the (020), (210), and (211) planes of an orthorhombic crystal lattice based on the antiparallel pleated β-sheet in ADF3^23^; the *d*-spacings were 0.51, 0.45 and 0.37 nm, respectively. The WAXD patterns of the β-sheet crystals of the L- and T-polyAs were similar, showing a shift for the peak of the (210) plane (*d* = 0.43 nm)^28,29^. The variation in the *d*-spacing of the (210) plane can be explained by the differences in the crystal lattice structures among the silks of different spider species^23,30,31^. As the L-polyA content increased, all of the peaks associated with polyA’s β-sheet became more intense, as shown in Fig. 5a. However, the peak intensity of the spider silk’s (210) plane was enhanced more than the peak intensity of the polyA’s (210) plane when the L-polyA content was greater than 5 wt%. This indicates that L-polyA tends to aggregate in the composite films and influence the crystal structure of the inherent spider silk β-sheet because of the similarity between their sequences. The formation of additional spider silk’ β-sheet crystals was induced by L-polyA, resulting in films more rigid and brittle than the silk-only film. An apparent increase in the amount of β-sheet crystals was also observed in the IR spectra of prestretched films (Figs 6 and S12). The intensity of the C=O stretching band derived from β-sheet at 1620 to 1640 cm^−1^ increased after the addition of L- and T-polyA, and the increase was more significant in films with a high additive amount (10 wt%). The peak at the amide I region (1600 to 1700 cm^−1^) was deconvoluted to analyze the composition of secondary structures (Fig. S13), and the results are summarized in Table S2. The β-sheet structure obviously increased by increasing the additive amount up to 10%, whereas β-turn structure decreased. The β-sheet formation was caused by prestretching, and the peak of β-sheet was more emphasized at a higher prestretching ratio (Fig. S14)^20,27^. By contrast, the addition of T-polyA increased the intensity of all of the peaks associated with polyA’s β-sheet in the WAXD profiles; however, the peak intensity of the spider silk’s (210) plane showed no significant change (Fig. 5b). These results indicate that the crystal of T-polyA was homogeneously dispersed in the composite films and showed less effect on the silk’s β-sheet because of the telechelic structure. Both T-polyA and the inherent silk sequence simultaneously crystalized in the film, resulting in high toughness at approximately 1 wt% loading of T-polyA. However, the excess T-polyA at loadings greater than 5 wt% resulted in a large amount of polyA crystals, which made the composite film brittle.

**Figure 7:**
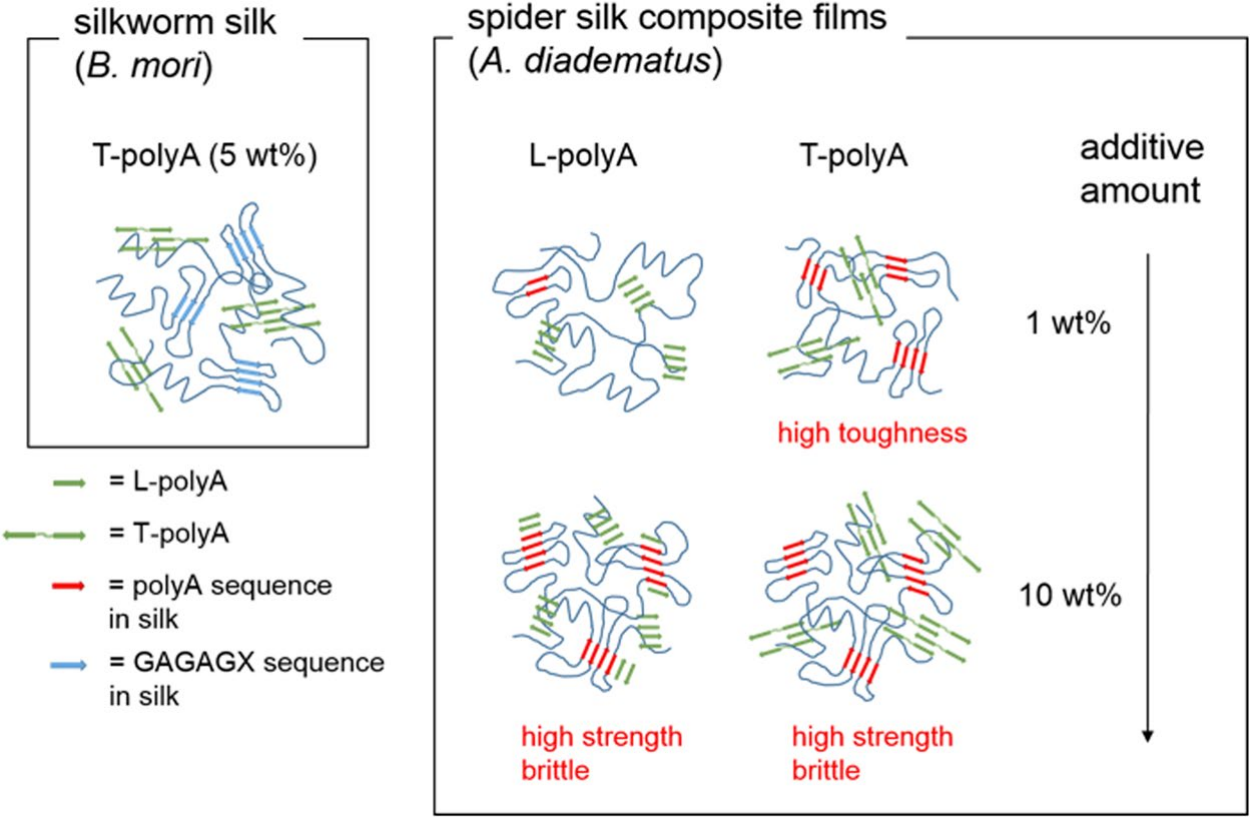
Illustration of antiparallel β-sheet structures formed in silk composite films doped with L- and T-polyA.

For the spider silk composite films doped with L- and T-polyA, the reinforcement of tensile strength was related with the formation of β-sheet crystal domains revealed by WAXD analysis. In the case of silkworm silk composite films in the previous work^3^, the addition of L-polyA into the silk provided the turbid films with a poor mechanical property, whereas the composite films with T-polyA (5 wt%) showed higher tensile strength and toughness compared to the silk-only film. T-polyA shows high tendency to assemble into fibrillar structures as revealed by AFM observation, but mismatches with the GAGAGX crystalline sequence in the silkworm silk (Fig. 7). Therefore, it was assumed that T-polyA formed β-sheet crystal domains independent of GAGAGX sequence, which resulted in the reinforcement of tensile property. In contrast, the addition of both L- and T-polyA in the spider silk provided transparent composite films with higher tensile strength than the silk-only film. Furthermore, the addition of T-polyA at a low additive amount (1 wt%) effectively enhance both the tensile strength and toughness, whereas the silkworm silk composite films containing T-polyA below 5 wt% exhibited no significant change in mechanical property. These results strongly indicate that the structural similarity between polyA additives and the crystalline sequence of the spider silk effectively facilitated the β-sheet crystal formation and thereby toughened the composite films. Adding higher amounts of dopants further developed β-sheet structures with different effects depending on the dopant structures. In the case of L-polyA, the β-sheet crystal of intact spider silk increased because L-polyA matches with the spider silk’s crystal in terms of similar length and sequence. In contrast, the composite films with high additive amount of T-polyA showed an increase in β-sheet structure derived from T-polyA. This indicates that the unique primary structure of T-polyA tends to exclusively assemble itself into the β-sheet crystals even coexisting with the analogous sequence of spider silk. Unfortunately, the composite films doped with the high amount of L- and T-polyA became rigid but brittle.

## Conclusion

Two-types of additives, L- and T-polyA, were used to prepare all bio-based composite films based on recombinant spider silk protein. The composite films exhibited tensile strength superior to that of the silk-only film. The differences in the primary structures of the polyA additives strongly affected the crystal structures of the composite films and thereby enhanced their toughness, which was supported by WAXD analysis. Structural similarity between polyA additives and the crystalline sequence of spider silk can enhance the ability to form β-sheet structures with great miscibility. As a result, an extremely low level of additive amount (1 wt%) could reinforced the tensile property of the spider silk composite films compared to silkworm silk composite films that have GAGAGX crystalline sequences. The polypeptide additives can be used not only to improve the physical property of silk materials including films and fibers, but can also offer novel functionalities.

## Materials and Methods

### Materials

The recombinant spider silk protein based on a sequence of a major ampullate spidroin ADF3 from *A. diadematus* with a His-tag was biosynthesized in *Escherichia coli*. Papain (EC No. 3.4.22.2) was purchased from Wako Pure Chemical Industries, Ltd. (Osaka, Japan) and used as received. The activity was approximately 0.5 U g^−1^, where one unit hydrolyzes 1 mmol of *N*-benzoyl-DL-arginine *p*-nitroanilide per minute at pH 7.5 and 25 °C. L-polyA was chemoenzymatically synthesized using papain accroding to the previous study^24,25^. The other chemicals were purchased from Tokyo Chemical Industry Co., Ltd. (Tokyo, Japan) and used as received without purification, unless otherwise noted.

### Construction of plasmid and strain

A gene encoding a part of the dragline silk spidroin ADF3 obtained from *A. Diadematus* (NCBI accession number: AAC47010) with His10-tag and human rhinovirus 3C protease cleavage sequence at the N-terminus was designed on a pUC57 vector and synthesized by GenScript Japan Inc. (Tokyo, Japan). The vector was treated with Nde I and EcoR I restriction enzymes and transfered to pET22b(+) vector. Site-selective mutagenesis on the pET22b(+) vector was performed using PrimeSTAR^®^ Mutagenesis Basal Kit (Takara Bio Inc., Otsu, Japan) to convert a codon GTG for 543Val to a stop codon TAA. The successful mutation was confirmed by sequencing using Applied Biosystems 3130xl Genetic Analyzer (Thermo Fisher Scientific Inc., Waltham, MA USA). Rosetta (DE3) strain of *E. coli* was transformed by the resulting gene on the pET22b(+) vector to obtain a recombinant strain.

### Synthesis of recombinant spider silk protein

A single colony of the recombinant strain was cultured on LB medium (2 mL) containing ampicillin for 15 h. An aliquot (1.4 mL) of the medium was inoculated on another LB medium (140 mL) containing ampicillin, and cultured at 37 °C and at 200 rpm until OD_600_ became 3.5. The resulting medium was transferred into a 2xYT medium (7 L) containing ampicillin. A solution of glucose (50 wt%, 140 mL) was added to the medium and the strain was cultured until OD_600_ became 4.0. Then, the production of the recombinant spider silk protein was induced by adding isopropyl β-D-1-thiogalactopyranoside (IPTG, final concentration of 0.5 mM) and the cultivation was continued for 2 h. The cells were collected by centrifugation. The resulting pellet was washed with Tris-HCl buffer (30 mL, 20 mM, pH 7.4) containing phenylmethylsufonyl fluoride (PMSF) in isopropanol (0.3 mL, 0.1 M), and the cells were dispersed in urea buffer (7.5 M urea; 10 mM NaH_2_PO_4_; 20 mM NaCl; 1 mM Tris-HCl, pH 7.0). The cells were disrupted by sonication, and the cell debris was removed by centrifugation. The supernatant was then dialyzed against Milli-Q water using a cellulose tube, and the precipitated protein was collected by centrifugation. The crude recombinant protein with a His-tag was purified using nickel affinity chromatography eluted with 6 M urea buffer. The eluted solution was dialyzed against Milli-Q water for 48 h using CE tube (MWCO: 3.5 kDa) and lyophilized to afford the recombinant protein powder.

### Synthesis of telechelic T-polyA via chemoenzymatic polymerization

A mixture of l-alanine ethyl ester hydrochloride (0.922 g, 6.0 mmol), telechelic type terminal modifier (compound **1** in Fig. S1, 0.190 g, 0.60 mmol), phosphate buffer (2.0 mL, 1 M, pH 8.0), and tetrahydrofuran (1.0 mL) were added to a 10-mL glass tube equipped with a stir bar and the mixture was stirred at 40 °C until all substrates were completely dissolved. Then, a solution of papain (0.300 g) in phosphate buffer (2.0 mL) was added in one portion. The final concentrations of alanine ethyl ester and papain were 1 M and 50 mg mL^−1^, respectively. The mixture was stirred at 60 °C and 800 rpm for 6 h using an EYELA ChemiStation (Tokyo, Japan). After cooling to room temperature, the precipitate was collected by centrifugation at 7000 rpm and 4 °C for 10 min. The crude product was washed twice with deionized water and methanol and lyophilized to afford a white powder. The yield was 0.266 g (75%). The polymerization was conducted using various organic solvents and at various temperatures for optimizing the yield, and the results are lised in Table S1.

### Fabrication of silk composite films doped with polyalanine motifs

Typical procedure of the fabrication of silk composite film is shown for the composite film containing 5-wt% T-polyA. Lyophilized spider silk protein (0.285 g) and T-polyA (0.015 g, 5 wt% of the total components) were completely dissolved in HFIP (2.7 mL, 10 w/v%) under stirring. The solution was gently poured into a Teflon petri dish with a diameter of 100 mm and dried in a fume hood at room temperature overnight. The film was peeled off of the dish and further dried in a desiccator under vacuum for 4 h. The final thickness of the film was ca. 75 μm. Silk composite films containing T- or L-polyA with different compositions (ranging from 0.5 to 15 wt% of the total components) were also fabricated using the same procedure.

### Tensile deformation test of the recombinant spider silk composite films

Three types of silk films (silk only, doped with T-polyA, and doped with L-polyA) were cut into small plates (3.5 mm × 15 mm). The plate samples were immersed in methanol for 5 min and slowly pre-stretched using a uniaxial film stretcher (IMC-1A11, Imoto Machinery Co., Ltd., Tokyo, Japan) until the samples reached a 1.25-, 1.5-, 1.75-, or 2-fold length (stretching ratio was 25, 50, 75, or 100%, respectively). The pre-stretched films were fixed on a glass petri dish using double-sided tape and dried in a desiccator at room temperature under vacuum for 3 h. Each pre-stretched film was placed on a load cell (500 N) by clamping at both edges, and the tensile properties were measured using an EZ-LX HS universal/tensile tester (Shimadzu Corporation, Kyoto, Japan) at a stretching rate of 0.5 mm min^−1^ (strain rate: 1.65 × 10^−3^ s^−1^) at 25 °C and 55 to 60% relative humidity. The tensile properties of the silk composite films (i.e., Young’s modulus, maximum tensile strength, elongation at break, and toughness) were calculated from the obtained stress-strain curves. Five replicates were performed for each measurement, and the values of the mechanical properties were averaged to determine the standard deviation. All the data of the composite films were subjected on a statistical analysis using an unpaired *t*-test vs the silk only film with a two-tailed distribution and differences were considered statistically significant at *p* < 0.05.

### Structural analyses

The infrared (IR) spectra of the bulk samples were recorded by a IRPrestige-21 Fourier transform infrared spectrophotometer (Shimadzu Corporation, Kyoto, Japan) with a MIRacle A single reflection ATR unit using a Ge prism. The ^1^H nuclear magnetic resonance (NMR) spectra were recorded by a Varian NMR System 500 (Varian Medical Systems, Palo Alto, CA) at 25 °C and at the frequencies of 500 MHz. Dimethylsulfoxide-*d*_6_ (DMSO-*d*_6_) with trifluoroacetic acid-*d* (TFA-*d*) (5/1 in volume) was used as the solvent for the polypeptides with tetramethylsilane as the internal standard. The synchrotron wide angle X-ray diffraction (WAXD) measurements of the silk composite films and polyA powdery samples were performed by a BL45XU beamline at SPring-8, Harima, Japan, using an X-ray energy of 12.4 keV (wavelength: 0.1 nm). For atomic force microscope (AFM) observations, polyalanines obtained by the chemoenzymatic polymerization were dispersed in methanol/water (50/50 v/v %, 1 mg mL^−1^) by sonication for 30 min at r.t. After incubating the dispersion at r.t. for 7 days, an aliquot (5 μl) of the supernatant was deposited on a mica substrate, then dried at r.t. for over 12 h. The AFM observations were performed by an AFM5300E (Hitachi High-Tech Science Corporation, Tokyo, Japan) in the dynamic force mode (topographic and phase modes) with an SI-DF3 cantilever (resonant frequency: 29 kHz, force constant: 1.9 N m^−1^) for the samples on the mica substrate.

## Electronic supplementary material

Supporting information
