# Supplementary material for: Spider dragline silk composite films doped with linear and telechelic polyalanine: Effect of polyalanine on the structure and mechanical properties
Source: Sci Rep. 2018 Feb 26;8:3654. doi: 10.1038/s41598-018-21970-1 (PMC5827030; doi:10.1038/s41598-018-21970-1)
Supplement: Supplementary file 1 — Supporting information [file 41598_2018_21970_MOESM1_ESM.pdf]

Supplementary Information

Spider dragline silk composite films doped with linear and telechelic polyalanine: Effect of polyalanine on the structure and mechanical properties

Kousuke Tsuchiya[1,*], Takaoki Ishii[1], Hiroyasu Masunaga[2], and Keiji Numata[1,*]

[1]Enzyme Research Team, Biomass Engineering Research Division, RIKEN Center for Sustainable Resource Science, 2-1 Hirosawa, Wako, Saitama 351-0198, Japan.
[2]Japan Synchrotron Radiation Research Institute, 1-1-1, Kouto, Sayo-cho, Sayo-gun, Hyogo 679-5198, Japan.

- Fig S1-S14
- Table S1-S2

Supplementary Figure S1. Amino acid sequence of recombinant spider silk protein based on ADF3 from *Araneus diadematus*. Periodically observed polyalanine sequences (more than three alanines) are shown in red.

```
MHHHHHHHHHHSSGSSLEVLFQGPARAGSGQQGPGQQGPGQQGPGQQGPY
1   5    10   15   20   25   30   35   40   45   50
GPGASAAAAAAGGYGPGSGQQGPSQQGPGQQGPGGQGPYGPGASAAAAAA
    55   60   65   70   75   80   85   90   95  100
GGYGPGSGQQGPGGQGPYGPGSSAAAAAAGGNGPGSGQQGAGQQGPGQQG
   105  110  115  120  125  130  135  140  145  150
PGASAAAAAAGGYGPGSGQQGPGQQGPGGQGPYGPGASAAAAAAGGYGPG
   155  160  165  170  175  180  185  190  195  200
SGQGPGQQGPGGQGPYGPGASAAAAAAGGYGPGSGQQGPGQQGPGQQGPG
   205  210  215  220  225  230  235  240  245  250
GQGPYGPGASAAAAAAGGYGPGYGQQGPGQQGPGGQGPYGPGASAASAAS
   255  260  265  270  275  280  285  290  295  300
GGYGPGSGQQGPGQQGPGGQGPYGPGASAAAAAAGGYGPGSGQQGPGQQG
   305  310  315  320  325  330  335  340  345  350
PGQQGPGQQGPGGQGPYGPGASAAAAAAGGYGPGSGQQGPGQQGPGQQGP
   355  360  365  370  375  380  385  390  395  400
GQQGPGQQGPGQQGPGQQGPGQQGPGQQGPGGQGAYGPGASAAAGAAGGY
   405  410  415  420  425  430  435  440  445  450
GPGSGQQGPGQQGPGQQGPGQQGPGQQGPGQQGPGQQGPGQQGPYGPGAS
   455  460  465  470  475  480  485  490  495  500
AAAAAAGGYGPGSGQQGPGQQGPGQQGPGGQGPYGPGAASAA
   505  510  515  520  525  530  535  540
```

Supplementary Figure S2. Chemoenzymatic synthesis of telechelic poly(L-alanine) (T-polyA) using papain.

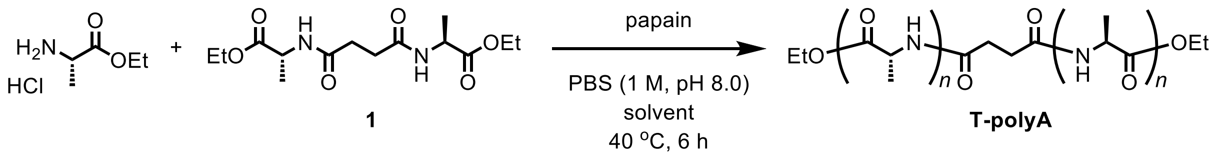

Supplementary Figure S3. Stress-strain curves of the recombinant spider silk films obtained at RH 58% with different pre-stretching ratios: (a) 0%; (b) 25%; (c) 50%; (d) 75%; (e) 100%. Five replicates were carried out for each experiment.

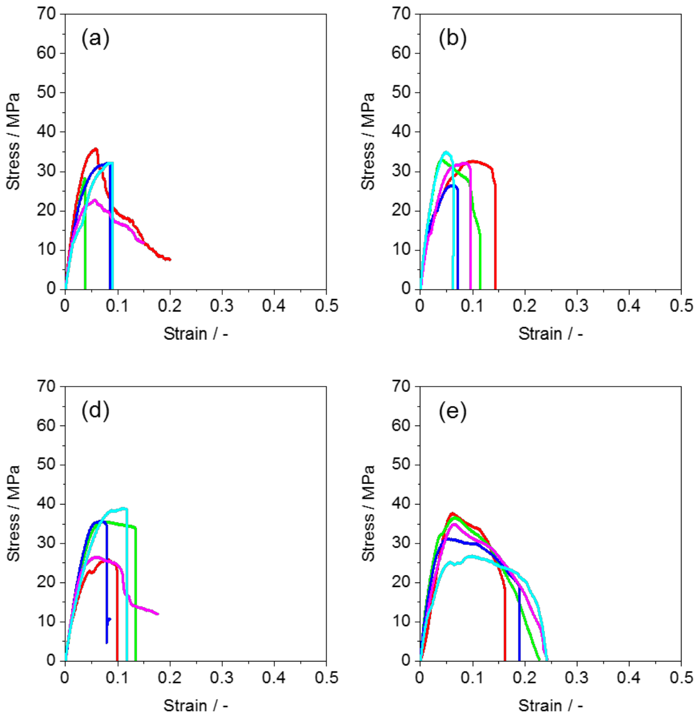

Supplementary Figure S4. Stress-strain curves of the composite films doped with 5 wt% T-polyA obtained at RH 58% with different pre-stretching ratios: (a) 0%; (b) 25%; (c) 50%; (d) 75%; (e) 100%. Five replicates were carried out for each experiment.

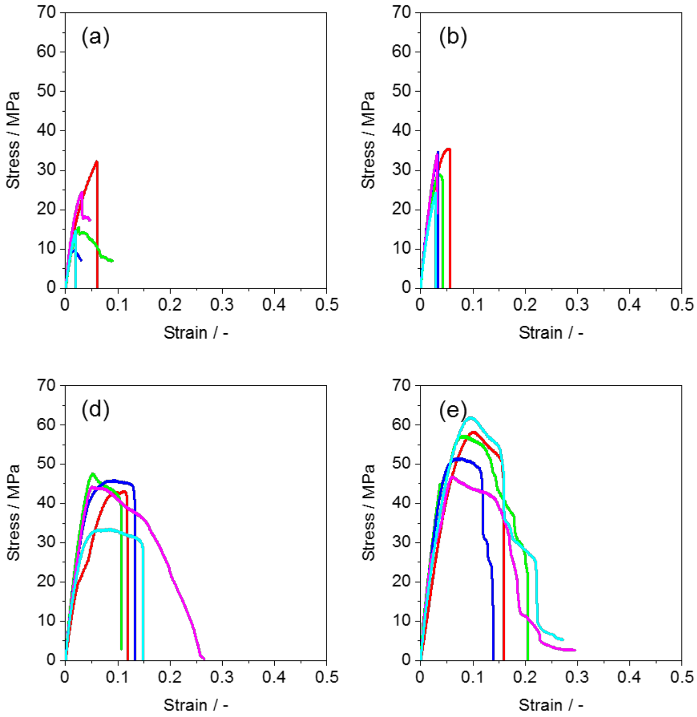

Supplementary Figure S5. Stress-strain curves of the recombinant spider silk films with pre-stretching ratio of 100% obtained at different relative humidity (RH): (a) RH 23%; (b) RH 43%; (c) RH 58%; (d) RH 84%. Five replicates were carried out for each experiment.

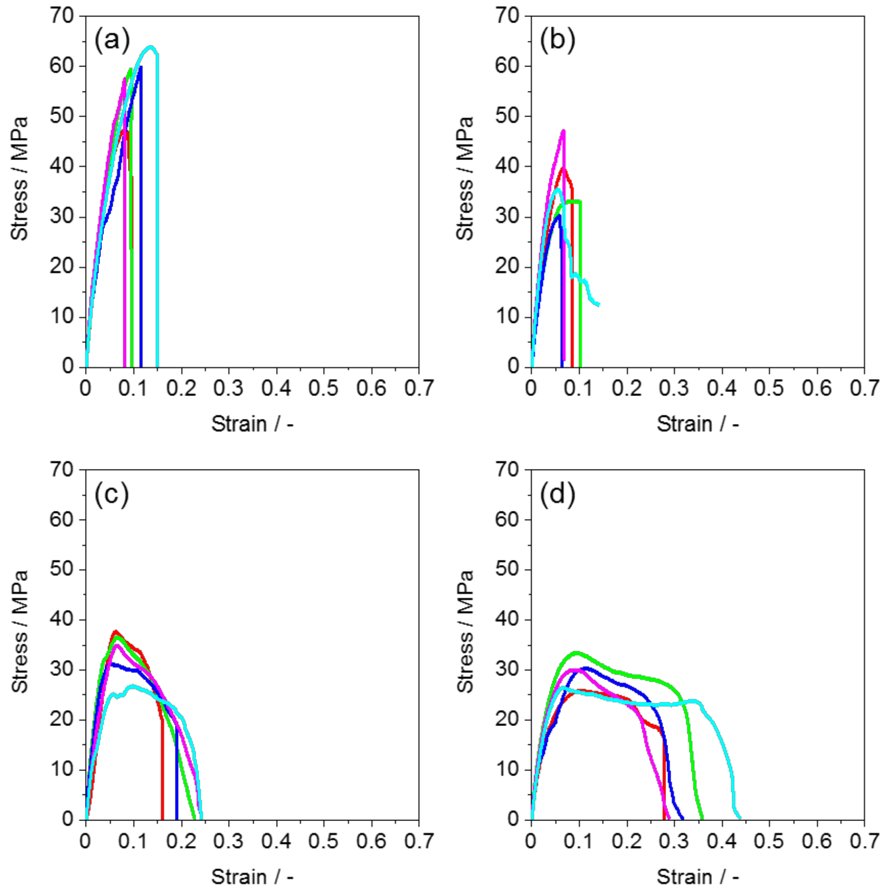

Supplementary Figure S6. Stress-strain curves of the spider silk composite films doped with 5 wt% of T-polyA with pre-stretching ratio of 100% obtained at different relative humidity (RH): (a) RH 23%; (b) RH 43%; (c) RH 58%; (d) RH 84%. Five replicates were carried out for each experiment.

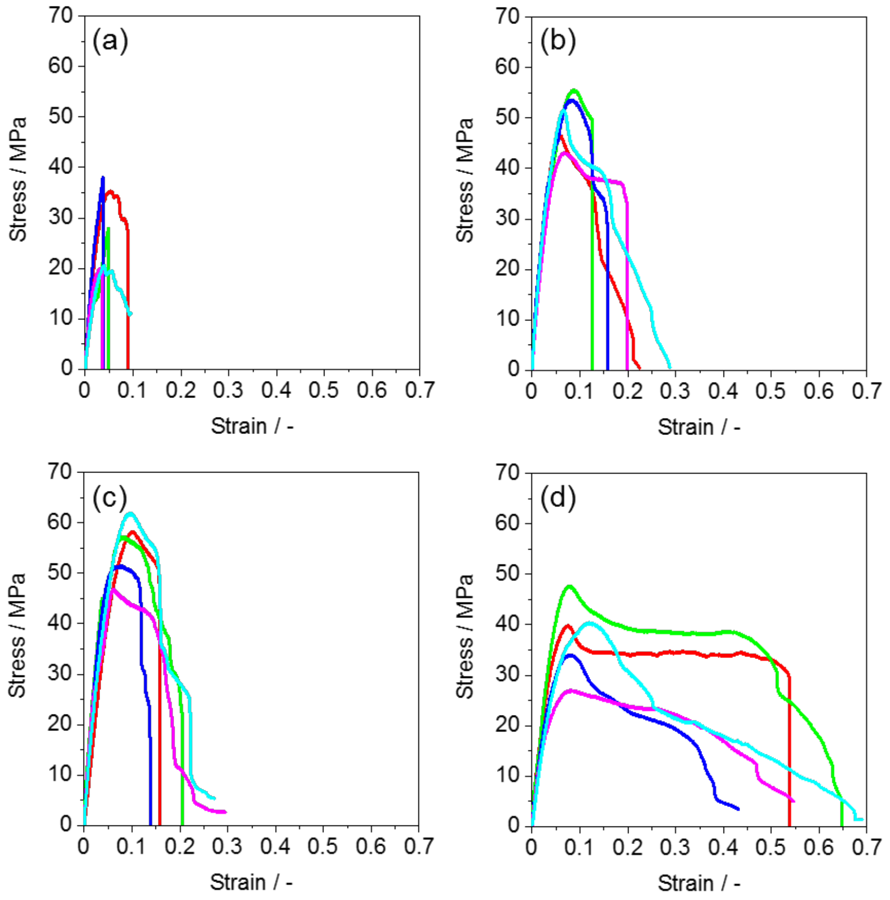

Supplementary Figure S7. Stress-strain curves of the spider silk composite films doped with (a) 1 wt%, (b) 2.5 wt%, (c) 5 wt%, (d) 7.5 wt%, (e) 10 wt%, and (f) 15 wt% of L-polyA with pre-stretching ratio of 100% obtained at RH 58%. Five replicates were carried out for each experiment.

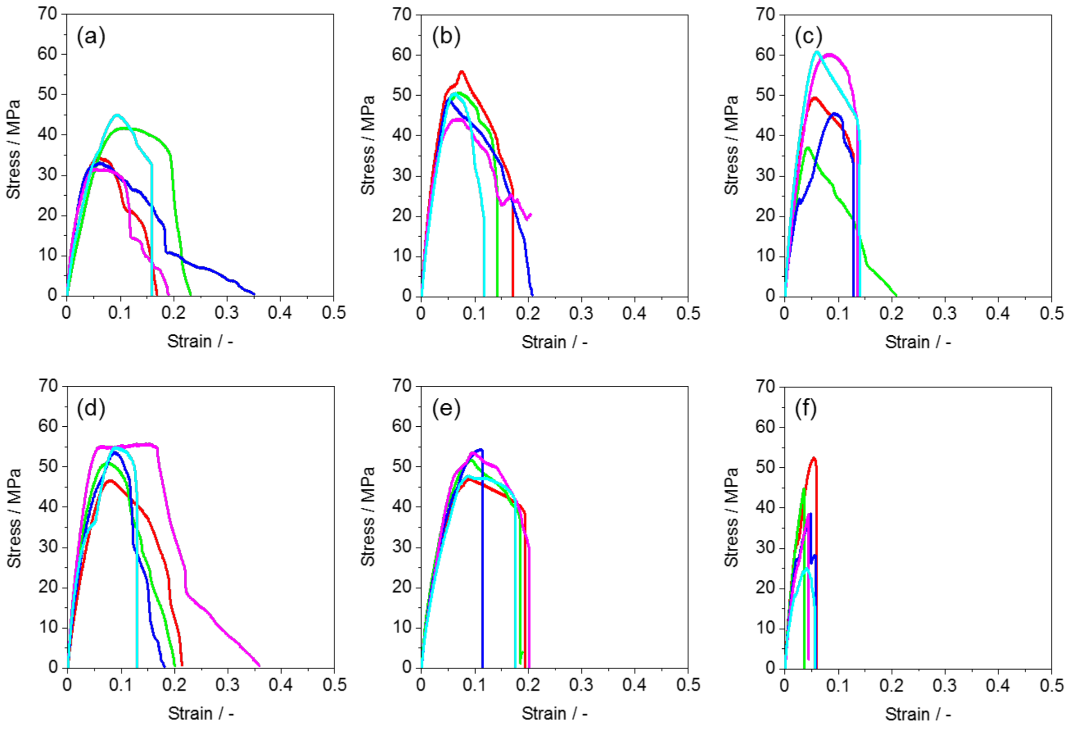

Supplementary Figure S8. Stress-strain curves of the spider silk composite films doped with (a) 0.5 wt%, (b) 1 wt%, (c) 1.5 wt%, (d) 2.5 wt%, (e) 5 wt%, and (f) 10 wt% of T-polyA with pre-stretching ratio of 100% obtained at RH 58%. Five replicates were carried out for each experiment.

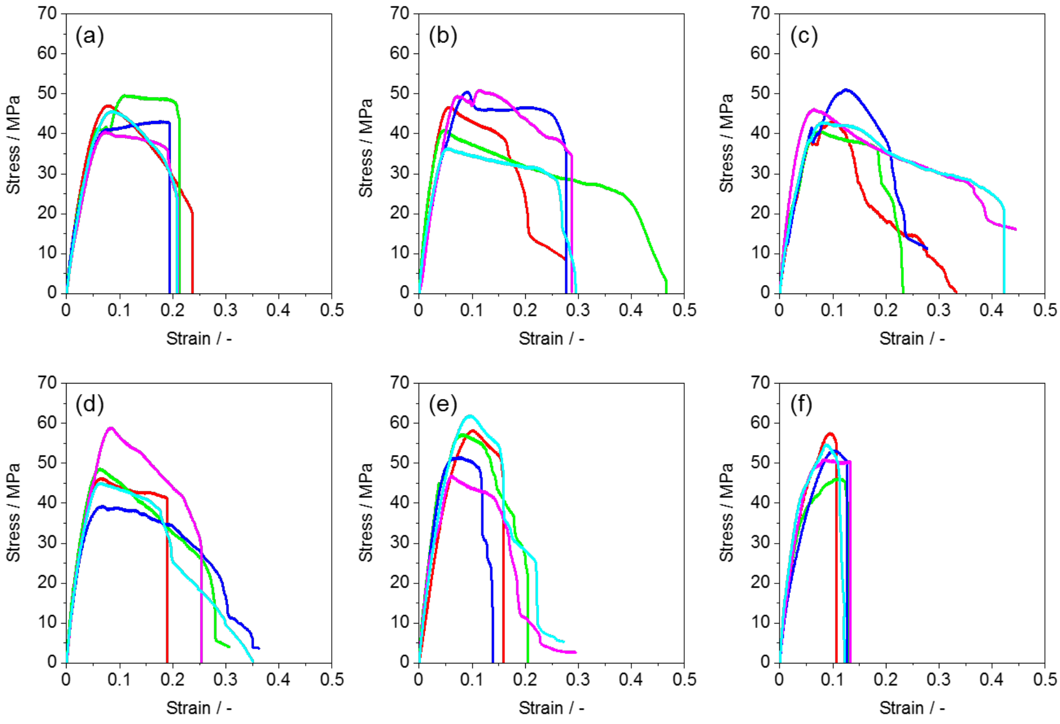

Supplementary Figure S9. WAXD 1D profiles of the composite films doped with L-polyA (a: 1 wt%; c: 10 wt%) and T-polyA (b: 1 wt%; d: 10 wt%) with various pre-stretching ratio (orange: 0%; red: 25%; green: 50%; blue: 75%; magenta: 100%). Black line shows the WAXD profile of powder L- and T-polyA samples.

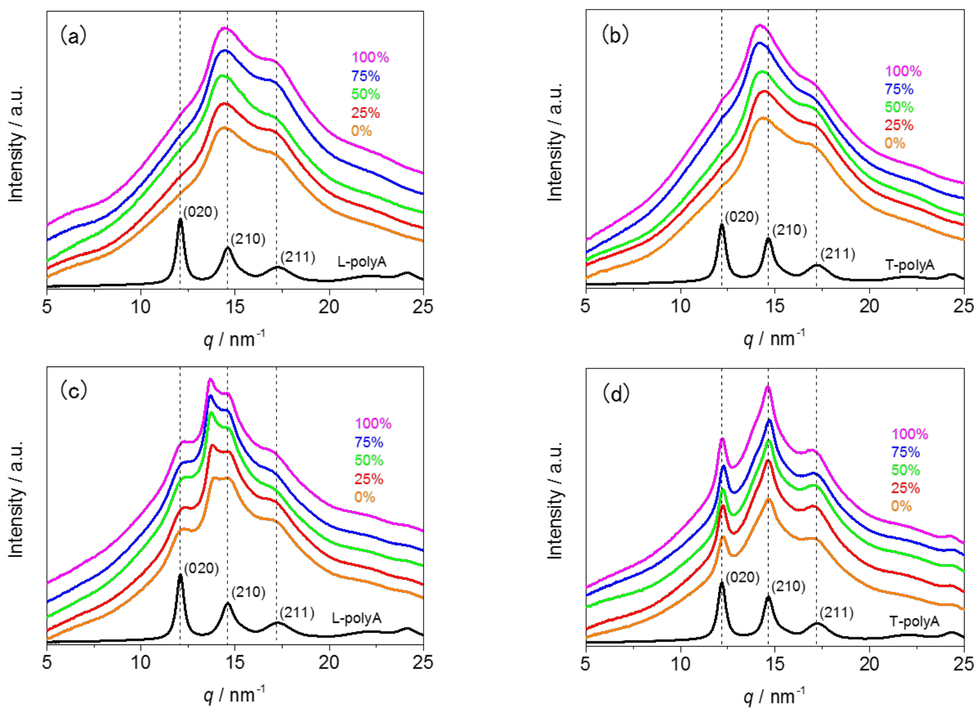

Supplementary Figure S10. WAXD 2D profiles of the silk composite films doped with L-polyA (a: 1 wt%; b: 2.5 wt%; c: 5 wt%; d: 10 wt%) with a prestretching ratio of 100%.

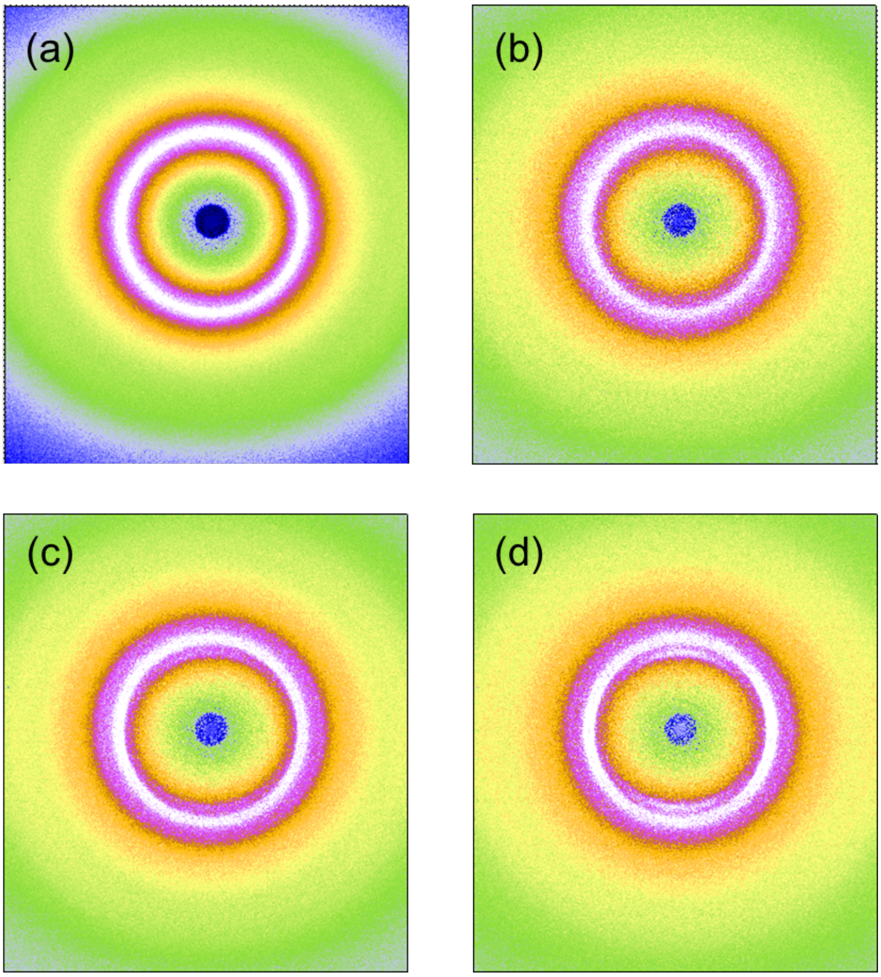

Supplementary Figure S11. WAXD 2D profiles of the silk composite films doped with T-polyA (a: 1 wt%; b: 2.5 wt%; c: 5 wt%; d: 10 wt%) with a prestretching ratio of 100%.

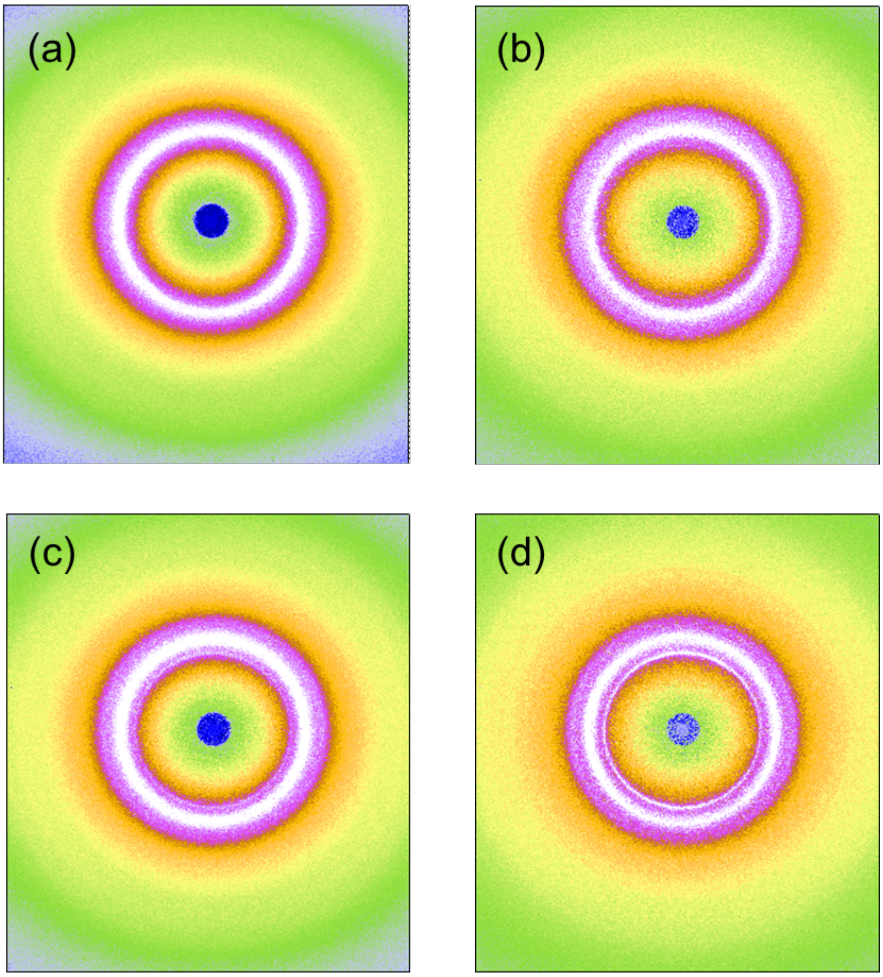

Supplementary Figure S12. IR spectra of the silk composite films doped with (a) L-polyA and (a) T-polyA with different additive amount (0, 1, 2.5, 5, and 10 wt%) at a 100 % prestretching ratio. Bottom profiles show the IR spectra of L- and T-polyA additives.

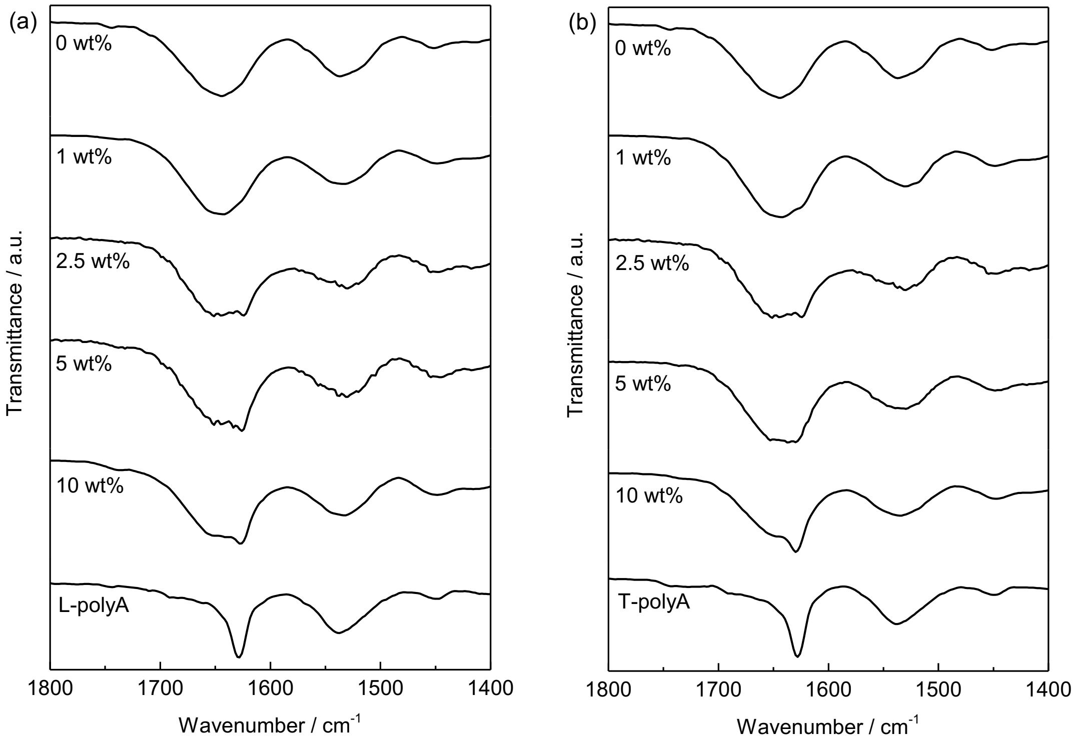

Supplementary Figure S13. Peak deconvolution of IR spectra of the composite films with (a) 1 wt% and (b) 10 wt% T-polyA in amide I region. Black and red solid lines are the original spectrum and a fitting curve, and the broken lines are deconvoluted peaks assigned to the secondary structures.

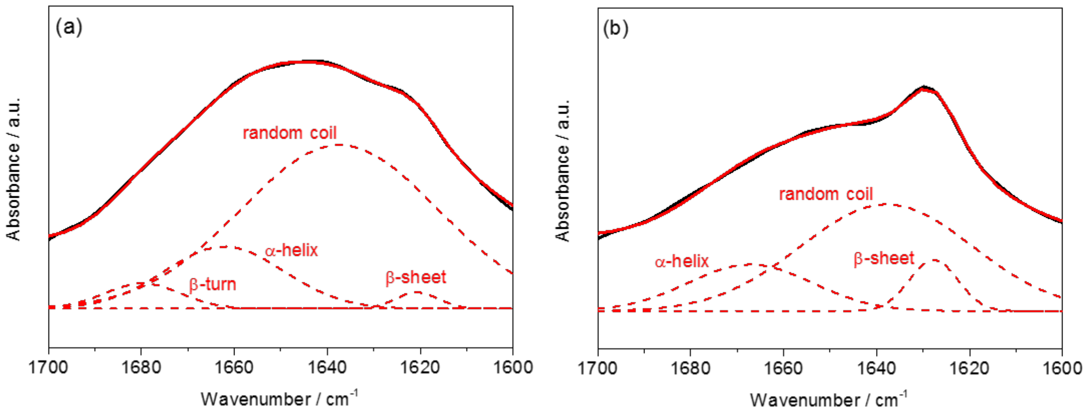

Supplementary Figure S14. IR spectra of the silk composite films doped with L-polyA (a: 1 wt%; b: 10 wt%) and T-polyA (c: 1 wt%; d: 10 wt%) with various prestretching ratios.

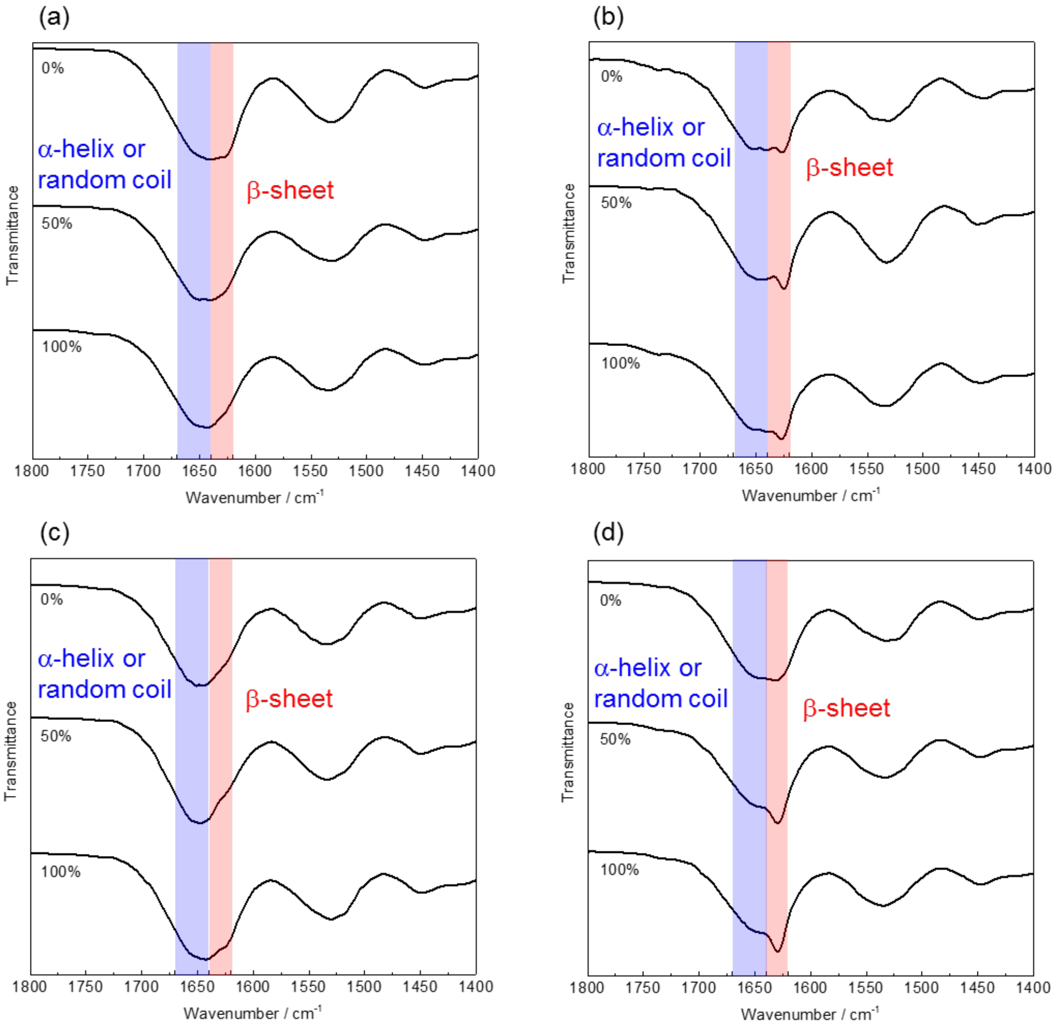

Supplementary Table S1. Chemoenzymatic synthesis of telechelic poly(L-alanine) (T-polyA) under various conditions.

Run[a]	Solvent	Temp (°C)	Yield[b] (%)	DP[c]
1	methanol	40	12	6.1
2	ethanol	40	33	6.3
3	chloroform	40	0	-
4	tetrahydrofuran	40	61	6.0
5	tetrahydrofuran	60	75	5.9

[a] Polymerization was carried out using papain (50 mg mL^{-1}) in 1 M phosphate buffer/organic solvent (5 mL/1 mL) at 40 or 60 °C for 6 h. [b] Precipitate was collected by centrifugation. [c] Degree of polymerization determined by ^1H NMR.

Supplementary Table S2. Composition of the secondary structures in the silk composite films estimated from IR spectra.

Additive	Loading amount (wt%)	Composition (%)[a]			
		β-sheet	random coil	α-helix	β-turn
None	-	0	69.2	18.3	12.5
L-polyA	1	0.3	77.7	16.6	5.4
	2.5	5.9	75.6	17.5	1.0
	5	6.9	80.9	12.2	0
	10	9.3	61.7	29.0	0
T-polyA	1	1.8	75.5	18.0	4.7
	2.5	3.0	89.6	6.5	0.9
	5	4.6	66.2	29.2	0
	10	9.5	68.5	22.0	0

[a] Determined by comparing the area of each deconvoluted peak at amide I region in the IR spectra of the silk composite films.
